# Numerosity Perception in Peripheral Vision

**DOI:** 10.3389/fnhum.2021.750417

**Published:** 2021-11-03

**Authors:** Min Susan Li, Clement Abbatecola, Lucy S. Petro, Lars Muckli

**Affiliations:** Centre for Cognitive Neuroimaging, School of Psychology and Neuroscience, University of Glasgow, Glasgow, United Kingdom

**Keywords:** numerosity perception, peripheral vision, spatial integration, computational modelling, psychophysics

## Abstract

Peripheral vision has different functional priorities for mammals than foveal vision. One of its roles is to monitor the environment while central vision is focused on the current task. Becoming distracted too easily would be counterproductive in this perspective, so the brain should react to behaviourally relevant changes. Gist processing is good for this purpose, and it is therefore not surprising that evidence from both functional brain imaging and behavioural research suggests a tendency to generalize and blend information in the periphery. This may be caused by the balance of perceptual influence in the periphery between bottom-up (i.e., sensory information) and top-down (i.e., prior or contextual information) processing channels. Here, we investigated this interaction behaviourally using a peripheral numerosity discrimination task with top-down and bottom-up manipulations. Participants compared numerosity between the left and right peripheries of a screen. Each periphery was divided into a centre and a surrounding area, only one of which was a task relevant target region. Our top-down task modulation was the instruction which area to attend – centre or surround. We varied the signal strength by altering the stimuli durations i.e., the amount of information presented/processed (as a combined bottom-up and recurrent top-down feedback factor). We found that numerosity perceived in target regions was affected by contextual information in neighbouring (but irrelevant) areas. This effect appeared as soon as stimulus duration allowed the task to be reliably performed and persisted even at the longest duration (1 s). We compared the pattern of results with an ideal-observer model and found a qualitative difference in the way centre and surround areas interacted perceptually in the periphery. When participants reported on the central area, the irrelevant surround would affect the response as a weighted combination – consistent with the idea of a receptive field focused in the target area to which irrelevant surround stimulation leaks in. When participants report on surround, we can best describe the response with a model in which occasionally the attention switches from task relevant surround to task irrelevant centre – consistent with a selection model of two competing streams of information. Overall our results show that the influence of spatial context in the periphery is mandatory but task dependent.

## Introduction

Visual resolution decreases toward the periphery of the visual field, compared to foveal vision. Accordingly, while functional brain imaging research using a visual occlusion paradigm shows that the content of a visual scene can be decoded from brain activity patterns in a non-stimulated, peripheral part of the retinotopic visual cortex (Smith and Muckli, 2010; Muckli et al., 2015; Revina et al., 2018; Morgan et al., 2019), human peripheral vision tends to generalise scene information, as evidenced by behavioural phenomena such as crowding (e.g., Balas et al., 2009), the uniformity illusion (Otten et al., 2017), and a higher prominence of gist processing (Larson and Loschky, 2009). This tendency to generalize and blend information is ecologically relevant, if we consider that one of the roles of peripheral vision is to monitor the environment for relevant changes while we use foveal vision to focus on the current task. For example, in rabbits, the upper peripheral visual field is particularly tuned for dark spots on blue skies signifying predator birds (Levick, 1967; Steele-Russell et al., 2012). As humans, we need to cancel out redundant or predictable information in the periphery to save processing power and not be too easily distracted. On the other hand, we need to be made aware of those changes in the environment that are sufficiently salient or unpredictable to be worth further consideration.

Here, we aimed to study behaviourally the specific perceptual processing supporting these features of peripheral vision. In particular, we were interested in whether these phenomena can be explained by a distinct calibration of bottom-up (i.e., sensory information) and top-down (e.g., Stewart et al., 2020) selection of task relevant visual space in the peripheral vision. We selected numerosity as a perceptual property because it is a low-level feature susceptible to gist processing (Park et al., 2016; Fornaciai et al., 2017), and independent to other primary visual properties like objects, colour, shape, or location (Burr and Ross, 2008). It is also easy to manipulate on a numerical continuum for psychophysics purposes (e.g., Valsecchi et al., 2013).

We designed a task using Maximum Likelihood Conjoint Measurement (MLCM; Ho et al., 2008; Knoblauch and Maloney, 2012; Maloney and Knoblauch, 2020), a signal-detection based scaling paradigm which we used to characterises the separate contribution of perceptual attributes to perceived numerosity in the periphery. We presented arrays of dots of varying numbers in the left and right peripheral visual fields and participants had to indicate whether there were more dots on the left or the right side. The peripheral areas on each side were further divided into a centre and a surrounding region, only one of which was the task-relevant target while the other was a task-irrelevant context. We could then quantify how perceived numerosity in the relevant part of the display (bottom-up information) is biased toward the number of dots presented in the irrelevant part (contextual information).

In the case that we found a perceptual bias toward the task-irrelevant signal, a possible account would be based on the imprecision of the top-down connections that span out to a larger region. The feedforward input is then not matched by the correct top-down predictions. In such a leaking model prediction errors around the boundaries could lead to an over or underestimation around the boundaries. This Predictive Coding account would lead to an integrative process in which bottom-up and top-down signals are combined to a distorted perception based on integration of a prediction error around boundaries (e.g., Rao and Ballard, 1999; Friston, 2005). An alternative outcome would be observing a serial process under which only one source of information can be perceived at a time (e.g., limited perceptual capacity; Yiǧit-Elliott et al., 2011), and including contextual signals increases the ambiguity of the overall stimuli to the extent that the irrelevant cue is sometimes perceived as the target (e.g., Craig, 1976; Berry and Fristedt, 1985). This model of a serial process could be explained in terms of Predictive Coding the way Jakob Hohwy explains binocular rivalry (Hohwy et al., 2008): the target model explains away the stimuli of the target region, but leaves the stimuli in the irrelevant regions unexplained as a consequence the irrelevant region creates so much prediction error that it sometimes forces the internal model into one that is consistent with the irrelevant information. In order to assess these two accounts, we consider the integration and switching models that make different assumptions about mandatory integration for perceiving the target and contextual stimuli. We aimed to clarify whether the perceptual decisions were made as a weighted average of relevant and irrelevant signals, or were made on the basis of a probability either according to the relevant or irrelevant part of the display, on a trial by trial basis.

Quantifying contextual effects also allowed us to study how the combination of perceptual cues is modulated by both higher-level top-down and bottom-up factors. Firstly, as a bottom-up factor, we varied the duration of presentation intervals to assess how contextual influence is related to the amount of acquired information. Summary representation of visual features in the periphery can be processed within a brief temporal window as short as 50 ms (Chong and Treisman, 2003), and here we aimed to investigate how perceived numbers in the periphery is affected by the strength of bottom-up signals with temporal intervals up to 1 s. Secondly, as a top-down factor, we modulated the area of display participants were attending to. We compared a condition in which the task-relevant targets were the centre areas and the irrelevant context were the surround areas (i.e., contextual filling-in from surround to centre), to a condition in which the task relevant targets were the surround areas and the irrelevant context were the centre areas (i.e., contextual expansion from centre to surround). By varying the spatial arrangement of task-relevant regions, we examine whether the perceptual mechanism of spatial interaction is comparable for filling-in or expanding the context information.

## Materials and Methods

### Participants

We recruited 18 volunteers with normal or corrected to normal vision (15 females and 3 males, mean age 25.3 ± 4.22 years) through the Psychology Experiments Participant Pool of the University of Glasgow, and we paid participants 6 pounds per hour. All participants gave informed consent prior to the experiment. We randomly assigned participants to two groups of equal size, each group performing a different task (see Procedure). The study was approved by the ethics committee of the College of Science and Engineering of the University of Glasgow and conducted according to the principles expressed in the Declaration of Helsinki.

### Apparatus and Design

The experiment was controlled by PsychoPy v3.2.3 (Peirce, 2007) on a Windows 10 HP EliteOne 800 All-in-one PC, with a monitor size of 525 × 296 mm, refresh rate of 59 Hz and 1,920 × 1,080 resolution. Participants were placed at a distance of 57.3 cm from the screen with a chin rest so that 1 cm was equivalent to 1 degree of visual angle.

**FIGURE 1:**
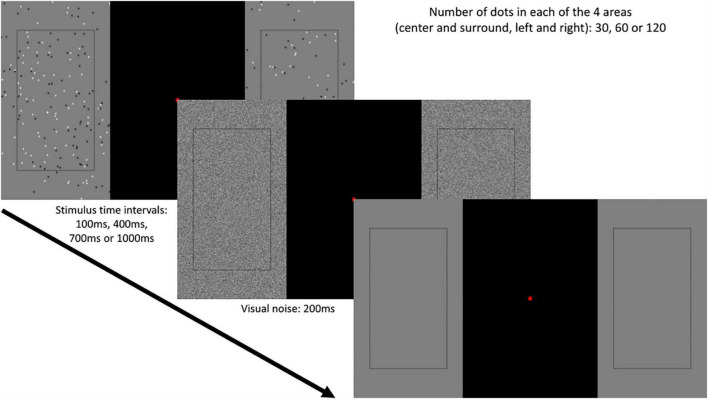
Experimental protocol. For each trial, a varying number of dots was presented in left centre, left surround, right centre, right surround, for a varying duration: 100, 400, 700 or 1,000 ms. This was followed by brief visual white noise, after which the observers had to discriminate, for the relevant area, either left or right side presented more dots (Two- Alternative Forced Choice).

The display had a mid-grey background with the central part of the visual field masked by a 200 × 295 mm black area, in order to restrict stimulations to only the near-peripheral visual field. A red fixation point (5 × 5 mm) was placed in the middle of the central black area. In the left and right peripheral parts (10 degrees each from the fixation point) were two peripheral displays, each divided into two sub-parts, *centre* and *surround*, with a black rectangle shape indicating the border of the centre (see Figure 1). The centre and surround areas were always of the same size for each peripheral display. Thus, the four regions of interest in the study were: centre (area inside the rectangle) in the left periphery (CL), centre in the right periphery (CR); surround (area outside of the rectangle) in the left periphery (SL) and surround in the right periphery (SR).

Visual stimuli were circular black and white dots (50 and 50%), 5 mm diameter each applied with a Gaussian blur. The spacing of dots was determined by a uniform random distribution. Note that the dots covered only the peripheral areas of the visual field, and not the central black area (Figure 1). In each trial, the number of dots in a given area (CL, CR, SL or SR) could be 30, 60 or 120, in accordance to the Weber’s Law (Fechner, 1860) that a multiplicative increase in the physical magnitude of numbers is expected to translate into a linear perceptual increase (Brannon et al., 2001; Ross, 2003; Jordan and Brannon, 2006; Merten and Nieder, 2009; Anobile et al., 2014). This yielded 9 possible combinations of the number of dots for the centre and surround regions on one side of the display (Table 1). Hence, for the whole display, there were a total of 36 combinations in which left and right peripheries were non-identical, as non-identical dot numbers on the two sides are required for the psychophysical task (see below). Centre/surround dot combinations were randomly assigned over left and right peripheral displays on each trial. In addition, we varied the duration of visual display by presenting the stimuli for 100, 400, 700 or 1,000 ms. We chose these values to include a range of exposure durations that gradually increase in clarity.

**TABLE 1:** An example of the experimental conditions (number of dots) presented on one side of the display.

**Experimental combinations**	**Centre**	**Surround**
	
	**Number of dots**	
1	120	30
2	120	60
3	120	120
4	60	30
5	60	60
6	60	120
7	30	30
8	30	60
9	30	120

### Procedure

The testing cubicle remained dark throughout the experiments to prevent observers from experiencing changes in luminosity. For each trial, participants were shown the stimuli (for 100, 400, 700 or 1,000 ms) followed by a 200 ms white visual noise (covering only both peripheral areas, excluding rectangles that define the central and surround areas and the mid-screen black area) to control for visual aftereffects. All observers performed a numerosity discrimination task: when the visual noise disappeared, observers had to press one of the two buttons to indicate whether there were more dots on the left or right relevant part of the screen. One of the two groups of participants was instructed that only the centre regions of both sides were relevant (judging CL vs. CR), and the other group was assigned the surround of both sides as relevant regions (judging SL vs. SR). The next trial began after a 300 ms inter-trial interval after each response. The experiment lasted approximately 30 min with a short break halfway through the experiment.

The 36 combinations of the number of dots were presented 8 times at each temporal interval, yielding 36 (combinations) × 4 (time intervals) × 8 (repetitions for each unique trial) = 1,152 trials in total for each participant. The timing factor was a within-subject design, and the task factor was a between-subject design to avoid adaptation effects and confusion between the tasks.

### Analysis

In this section we describe the implementation of Maximum Likelihood Conjoint Measurement, allowing us to use scaling measures to estimate the perceptual bias of judgments and examine the possibility of integrated perceptual information with three decision models. We also simulate the observer’s responses with two specific decision rules to determine the mechanism of such perceptual integration.

#### Maximum Likelihood Conjoint Measurement

Our protocol and analyses followed the principles of Maximum Likelihood Conjoint Measurement (MLCM; Knoblauch and Maloney, 2012; Maloney and Knoblauch, 2020), a signal-detection based scaling paradigm, under which the contribution of different features to perceptual decisions can be quantified. Although initially designed to study how multiple physical properties (e.g., visual roughness and glossiness) interact in their perception (Ho et al., 2008), MLCM has recently been applied to study how the properties of a background surface affect the perception of a central surface (Hansmann-Roth and Mamassian, 2017; Hansmann-Roth et al., 2018). Here, we manipulate the physical properties of centre and surrounding areas in the periphery, and we examine how irrelevant areas contribute to the perceived numerosity in the relevant area, depending on the task (centre task or surround task).

Assuming we are handling the data of an observer from the centre task group (CL vs. CR), the simplest decision model would be one where the observer compares some internal function of the number of dots in left and right centre areas:


(1) $$\Delta_{C}=\psi_{C}\,\left(C\,L\right)-\psi_{C}\,\left(C\,R\right)+\epsilon$$

Where CL and CR are the number of dots in the centre on the left and on the right, respectively. On a given trial, *ψ*_*C*_ is some internal function determining the perceived number of dots in centre on a single side given the actual number, ϵ is an unbiased and normally distributed decision noise, and *Δ*_*C*_ is the decision variable whereby the left side (if *Δ*_*C*_ > 0) or the right side (if *Δ*_*C*_ < 0) is chosen by the observer as containing the highest number of dots in the central area. This is called an Independence Model, and such a model assumes that the perceived number of dots in CL is completely independent from SL. However, it is also possible that the number of dots in SL will contribute to the numerical estimate of CL.

The simplest model to take such effects into account is the Additive Model:


(2) $$\Delta_{C}=\left[\psi_{C}\,\left(C\,L\right)+\psi_{S}\,\left(S\,L\right)\right]-\left[\psi_{C}\,\left(C\,R\right)+\psi_{S}\,\left(S\,R\right)\right]+\epsilon$$

Where SL and SR are the number of dots in surround on the left and on the right, respectively, and *ψ*_*S*_ is some internal function determining the contribution of the number of dots in the surround to the number of dots perceived in the centre on a single side. In the Additive Model, we make the hypothesis that the contribution of CL will not vary when changing the number of dots in SL (and vice versa).

To test this hypothesis, we can introduce interaction effects in the Full Model:


(3) $$\Delta_{C}=\left[\psi_{C}\,\left(C\,L\right)+\psi_{S}\,\left(S\,L\right)+\psi_{C\,S}\,\left(C\,L,S\,L\right)\right]-\left[\psi_{C}\,\left(C\,R\right)+\psi_{S}\,\left(S\,R\right)+\psi_{C\,S}\,\left(C\,R,S\,R\right)\right]+\epsilon$$

Where *ψ*_*CS*_ is a function determining interaction effects for each combination of the number dots in centre and surround. A possible instance of the full model could be a contrast enhancement model allowing for the centre to appear more numerous in the context of low numerosity and less in case of a surround of higher numerosity.

The three models defined here can be formalized as Generalized Linear Models to estimate ψ functions using maximum likelihood. As the models are nested within each other, the difference of their log-likelihoods is distributed as χ2 with degree of freedom the difference in the number of parameters (see e.g., Wood, 2015). We can therefore compare them to test our hypotheses using likelihood ratio tests (Maloney and Knoblauch, 2020). Such analysis, applied to different tasks (centre task group or surround task group) and different temporal intervals, will allow us to reconstruct different but comparable contribution values for perceiving CL, CR, SL, and SR.

#### Simulated Observers: Integration and Switching

If the independence model is rejected in favour of the additive model in MLCM, one would usually assume that results suggest a form of perceptual integration took place between the cues of interest. In our paradigm, for example, the number of dots perceived on one peripheral display can be interpreted as a weighted sum of the number of dots presented in the centre and surround of that side. However, because observers were instructed to judge only one of the two areas of each side, the perceptual weights of each sub-part contribution will vary depending on the task. For the centre task condition, for instance, more weight should be attributed to the centre and less to surround. This combination rule can be expressed as:


(4) $$\psi_{1}=w_{C}\cdot \psi_{C}\,\left(C\,L\right)+\left(1-w_{C}\right)\cdot \psi_{S}\,\left(S\,L\right)$$

**FIGURE 2:**
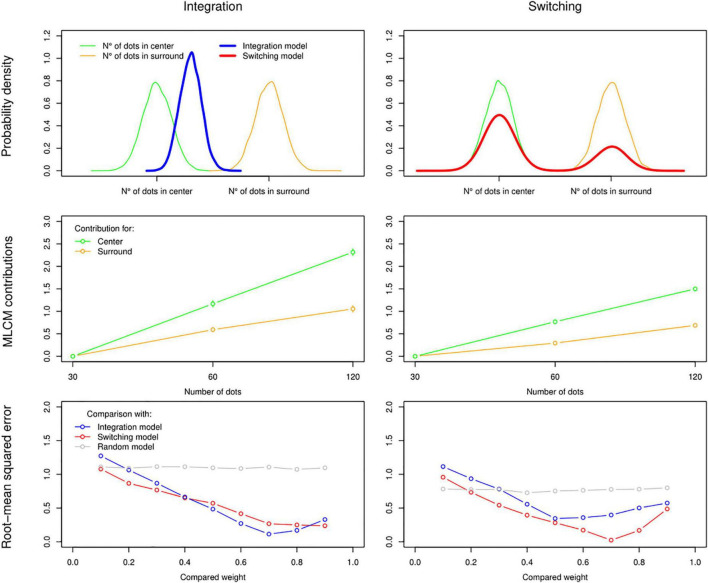
Simulating observers using an Integration rule (left column) or a Switching rule (right column) to estimate perceived numerosity in the centre and surround. Top panel: Probability density functions over 1,000 simulations of the same single combination. In this example weights of 0.7 for centre and 0.3 for surround are applied. Density for the perceived number of dots in centre and surround are represented in green and orange, respectively, and the resulting representations under Integration and Switching rules are in blue and red, respectively. Second panel: results of the MLCM analysis (additive model) applied to simulated responses of observers using Integration and Switching rules (in both cases, the simulated trials were the sum of all trials done by 9 of our human participants to make it comparable to empirical results). Bottom panel: comparison of two rules using RMSE between the simulated data (from the mid-panel) and other simulations with varying weight values and combination rules (Integration, Switching, and Random choice for each trial as a control).

Where *ψ*_1_ is the number of dots perceived on a single side of the screen and *w*_*C*_ ∈ [0,1] is the weight attributed from the centre. Under this rule and given assumptions from the Signal Detection Theory (Green and Swets, 1966), an experimental combination repeated over many independent trials with constant number of dots in centre and surround should follow a Gaussian distribution (Figure 2, top left panel).

As an alternative account, it is also plausible that responses followed a switching pattern: in any given trial, only one source of information is selected with probability *w_C_* of choosing according to centre and *1-w_C_* of choosing according to surround. This switching rule can be expressed as:


(5) $$\psi_{1}=\left[x<w_{C}\right]\cdot \psi_{C}\left(CL\right)+\left[x>w_{C}\right]\cdot \psi_{S}\left(SL\right)$$

Where *x* ∈ [0,1] is a random uniform variable. With the switching rule, an experimental combination repeated over many trials with constant number of dots in centre and surround should follow a bimodal distribution (Figure 2, top right panel).

Note that when weight values are extreme, i.e., *w*_*C*_=0 and *w*_*C*_=1, there is no difference between the two rules over many repetitions of the same trial. The average value over many repetitions with a constant *w_C_* is also the same between the rules.

We can then simulate the responses to an MLCM experiment with either integration rule, where the response to each trial is determined by:


(6) $$\Delta_{\text{I}}=w_{\text{C},\cdot }\,\left(C\,L-C\,R\right)+{\left(1-\,w_{\text{C}}\right)}_{\cdot }\,\left(S\,L-S\,R\right)+\epsilon$$

or switching rule, where the response to each trial is determined by;


(7) $$\Delta_{\text{S}}={\left[x<w_{\text{C}}\right]}_{\cdot }\left(CL-CR\right)+{\left[x>w_{\text{C}}\right]}_{\cdot }\left(SL-SR\right)+\epsilon$$

Where the Δ_*I*_ and Δ_*S*_ are the decision variables for the additive integration and switching model, respectively, and the notation otherwise follows Eqs. 2, 4, and 5. In particular we define the link functions as *ψ*_*C*_(*X*) = *X* and *ψ*_*s*_(*X*) = *X* for these simulated observers.

This yields contribution scales (Figure 2, middle panel) that are similar to typical empirical results when applying MLCM analysis. Most importantly, this method allows us to recover specifically which rule and weight value were implemented by a given simulated observer if we compare, with root-mean squared error, the observer’s result with the results of other simulated observers using a representative sample of rules and weight values (Figure 2, lower panel, which also includes a random rule under which the observer responds left or right randomly regardless of trial). We will use this method to determine which rule better describes the responses of our human observers under different experimental conditions.

## Results

### Maximum Likelihood Conjoint Measurement

First, we compared MLCM models in terms of complexity. We fitted the independence, additive and full models at each time interval for each participant. This allowed us to compare independence vs. additive and additive vs. full model in each case using likelihood ratio tests. The details of these comparisons are presented in Supplementary Table 1. To summarize, while the finding is somewhat noisy at 100 ms time interval due to the difficulty of the task, with longer time intervals we found that the independence model should always be rejected in favour of the additive model (all *p* < 0.001), and that in most cases the additive model should not be rejected in favour of the full model (except one participant from the surround task group at 400 ms: *χ*^2^(4) = 14.87, *p* = 0.005).

**FIGURE 3:**
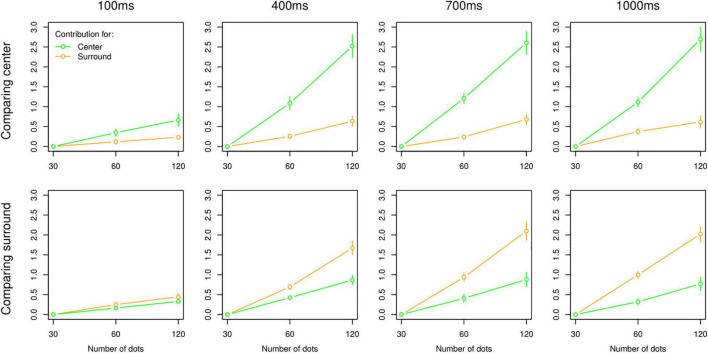
Results of MLCM analysis (additive model). Average contributions to the perceived numerosity in centre (green) and surround (orange) as a function of 30, 60, and 120 dots. Plots are organized by task (i.e., top panel: centre task, bottom panel: surround task) and presentation intervals (columns). All error bars are standard error of the mean.

This being the case, we turned our focus to the additive model, of which the average contributions across participants are shown in Figure 3. Given the outcome of model comparison, results of the additive model were expected, which showed a qualitative difference between 100 ms and other temporal intervals (i.e., 400, 700 ms, and 1 s). For the 100 ms interval, the contributions of centre and surround were always low across all combinations but not completely flat, which indicated that participants did not respond randomly even with 100 ms. For longer intervals, results showed more contribution of the task-relevant area. Interestingly, there was no reduced contribution of the task-irrelevant area with longer intervals, and these contributions were still significant even at the 1,000 ms interval for both centre and surround tasks as indicated by model comparison.

Moreover, the increase of the perceived number of dots when multiplying the actual number of dots in centre or surround by 2 is, as expected, relatively linear in all cases. The variation between timings and tasks can therefore be interpreted in terms of changes in perceptual weights and/or in perceptual strategy between Integration and Switching. These questions will be addressed in the next section.

In Supplementary Figure 1, we propose an alternative representation of the results without relying on MLCM modelling. The proportion of times each combination of centre/surround dot number was chosen for both tasks is represented for each time interval. Grey lines represent “ideal observers” responding either randomly (horizontal line) or always choosing the highest number of dots in the relevant dimension and randomly when the same number is presented on both sides (this occurs in 1/3 of cases). This shows that the number of dots in the relevant area (centre or surround) is the most important factor determining the observers’ choices, while the number of dots in the irrelevant area biases this choice. When the number of dots in the irrelevant area is 30 or 120, the number of dots in the relevant area is underestimated or overestimated, respectively. When the number of dots in the irrelevant area is 60, the estimated number of dots in the relevant area is very close to an ideal observer which responds to the number of dots in the relevant area with maximal accuracy. Compared to this analysis, MLCM allows a straightforward significance test for the effect of the irrelevant area (independent vs. additive model) and for interaction effects (additive vs. full model). It also allows further modelling of the underlying decision processes as proposed in the next section.

### Integration and Switching, Simulated Observers

**FIGURE 4:**
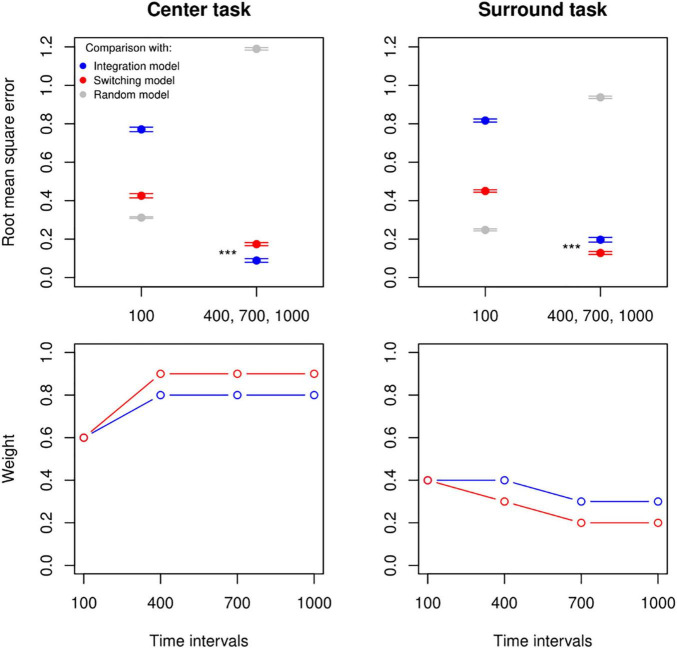
Observer simulations and optimal weights. Top panel: a comparison using the root-mean squared error between empirical data from the centre and surround conditions (left and right plot, respectively) and simulated data with varying combination rules (Integration in blue, Switching in red, and Random choice in grey). Ten datasets were simulated for 9 weight values (from 0.1 to 0.9) and for each rule. In both plots the lowest RMSE values across all weights at the 100 ms interval for all combination rules (and their standard errors across the 10 simulations) are shown on the left, while the mean and standard errors of the lowest RMSE values averaged across the remaining intervals are shown on the right. Bottom panel: pattern of weight values yielding the lowest RMSE at each timing and for Integration Model (in blue) and Switching Model (in red) when compared to empirical data (left: centre task, right: surround task). ***p < 0.001.

We compared the empirical data to simulated data to establish the best-fitted weight value and combination rule for each task and at each timing interval. In accordance with our previous results, we found a difference between the 100 ms interval and the other intervals. At 100 ms, neither the Integration nor Switching rule at any weight value performed better than an observer choosing at random to predict the participants’ decisions (Figure 4 top panel: the left tab in both plots). For the remaining longer intervals, we observed a consistent pattern in which the Random Choice Model was the worst-fitted model, the Integration Model was better at predicting the choices of observers judging according to centre, and the Switching Model was better at predicting the choices of observers judging according to surround (Figure 4 top panel, the right tab in both plots). This difference was significant over 10 independent simulations as assessed by independent *t*-tests: for centre task, Integration (*M* = 0.09, SD = 0.03), Switching (*M* = 0.17, SD = 0.03), *t*(18) = −6.85, *p* < 0.001. For Surround task, Integration (*M* = 0.20, SD = 0.04), Switching (*M* = 0.13, SD = 0.02), *t*(18) = 4.87, *p* < 0.001.

Furthermore, because each rule model had a single free parameter that has been varied to show the range of patterns that can be captured by the model (i.e., Figure 2), here with the smallest root-mean square error, we obtained the statistically optimal weight values for each timing interval and for each rule (Figure 4, bottom row, see Supplementary Table 2 for a statistical comparison between RMSE distributions for the best and second-to-best weight values at each time interval, and Supplementary Figure 2 for an illustration). Our results, again, suggested a consistent and robust behavioural trend that performances of 100 ms intervals were significantly distinctive from other longer intervals, where Integration and Switching Model performed similarly and obtained a weight value around 0.6 for the centre task and 0.4 for the surround task. It is worth noting that intervals above 100 ms held the same weight value for the centre task, but for the surround task there was an increase in optimal weight, specifically in a way that decisions favoured the relevant area with longer intervals until a floor effect.

## Discussion

We investigated the processing of numerical magnitude in peripheral visual displays, in which we found that the perceived numerosity of the target area is biased toward the number of dots presented in irrelevant neighbouring areas. Specifically, our results suggested that numerical magnitudes in the periphery were sampled following either a “weighted integration” or a probability switching’ process between the target and irrelevant areas. In other words, contextual cues presented in the surroundings were used for inferences about the numerosity in the centre; whereas contextual cues presented in the centre competed on a trial-by-trial basis to the perceived numerosity in the targeted surround. Thus, we argue that top-down factors, such as directing attention toward different areas in the peripheral visual field, have an impact on how predictions are incorporated into perceptual decisions about numerosity in peripheral vision.

We generally observed that numerosity perception in peripheral displays required a sufficient sampling time. Results from both centre and surround tasks showed that peripheral displays presented for 100 ms seemed highly ambiguous, and consistent with random responses of simulated observers. For presentation intervals longer than 100 ms, our results suggested an involuntary perceptual bias between task relevant and irrelevant parts of a display, supported by the significant advantage of the MLCM additive model (where the model’s decisions were based on both relevant and irrelevant areas), compared to the independence model (where the model considered only the relevant area). Interestingly, results showed that the perceptual contribution of target and irrelevant areas remained consistent across all intervals above 100 ms in the centre task, while the contribution of irrelevant central information while judging the surrounding region decreased progressively with longer intervals. By comparing the results of centre and surround task to the simulated observers, we identified an integration process for the centre task and a switching process for the surround task.

Specifically, perceived numerosity of a target region with irrelevant surrounding influences is best described as “contextual leaking in” effect, i.e., perceived numerosity of the central area is a perceptual combination with a weight of 0.8 attributed to the number of dots physically presented in centre, and 0.2 to the number of dots physically presented in its surround. In other words, it is as if the integration window fits with some insufficient precision on the centre areas, and 20% of the information from the surrounding spills into the decision in a way that the two channels cannot be separated with sufficient spatial precision. These weight values are stable across timing conditions above 100 ms, suggesting a common mechanism of integration for stimuli presented briefly and for longer durations. This low-level spatial integration involves incorporating a small amount of sensory signal from the surround area, and corresponds to an integration process described in the cue combination literature whereby independent noisy sources of information are combined into a weighted average, boosting the precision of perceptual estimates (e.g., Ernst and Banks, 2002; Ernst and Bülthoff, 2004; Hillis et al., 2004; Acerbi et al., 2014). Sensory integration, in fact, has been the only interpretation for additive MLCM models so far.

In contrast, perceptual decisions about surrounding areas with irrelevant central influences are described as an expansion contextual prior. In this case, participants’ responses are most consistent with a perceptual switching process, i.e., participants responded inaccurately according to the central region in some instances, while the surround targets are perceived accurately in the remaining majority of trials. Contrary to the weighted combination of perceptual inputs described above, this switching process does not require spatial integration of signals from relevant and irrelevant areas. Our results showed that participants made incorrect switches 30% of the time with 400 ms intervals, but the proportion was reduced to 20% of the trials for 700 and 1,000 ms intervals. To put this more parsimoniously, perhaps two separate streams of information are processed for the surround task, and the perceptual decision is reached by a lateral inhibition process between the two channels. Unlike an integration process, the switching process was therefore sensitive to changes in interval lengths, although with a floor effect. One may argue that a perceptual switching process is evidence for sequential processing with limited resources (e.g., Landy et al., 2007; Whiteley and Sahani, 2008; Scharff et al., 2011; Yiǧit-Elliott et al., 2011). This account implies that participants only had time to process one of the two parts of the display due to capacity limitations (e.g., difficulty of the surround task), where participants mistakenly prioritised assessing the number of dots in centre, instead of surround, for a small proportion of the trials. This account is partially supported by our empirical data where we showed a decreased tendency to make incorrect switches when participants were provided longer temporal intervals with the display. However, the fact that we still observed the switching behaviour at the longest interval makes this explanation less plausible unless we consider the possibility of another, incompressible, source of error such as motor or attentional mistakes.

**FIGURE 5:**
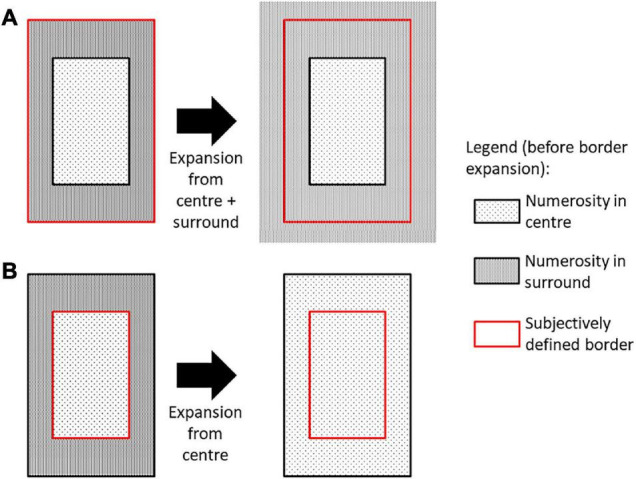
One plausible explanation for border expansion responses when participants were asked to judge the number of dots in the surrounding portion of the display. Here bistable perception is assumed to depend on whether the subjectively defined border which is expanded includes both central and surround areas, which doesn’t affect numerosity perception in surround **(A)**, or only the central area, which would then be expanded into the surround **(B)**.

A potential mechanism that could induce such an attentional error is biased bistable perception of ambiguous stimuli (e.g., Mamassian and Landy, 1998; Meng and Tong, 2004), in which there is a competition for awareness between several mutually exclusive interpretations of the same stimulus. Bistable perception could be influenced by the boundary extension effect, under which our recollection of scenes tends to extend beyond the border of what was actually presented (as reviewed in Hubbard et al., 2010). It shows how overall perception is affected by ambiguity in our displays. While boundary-extension has mostly been studied for natural scenes, line-drawing paradigms also provided evidence for such an effect (Gagnier and Intraub, 2012) and occurs also in the absence of semantic associations (McDunn et al., 2014), which leads to the possibility of similar processing for the current paradigm. If the display boundary is subjectively defined and the definition varies from trial to trial (e.g., condition to saliency of varying density of dots), such ambiguity would affect the perceived numerosity of the display. In particular, if the boundary includes the end of the complete peripheral display (centre *plus* surround, left or right), extending this “overall boundary” only affects the broader surrounding area. As a result, perceived numerosity in the surround would not be affected by this extension (see Figure 5A). However, if one considers only the boundary of the centre region, applying boundary extension would have the consequence of generalizing it to the surround area, making the observer perceive the same numerosity in centre and surround (Figure 5B), which may be the cause of incorrect switching in our results.

On the whole, we showed that perception of a task-relevant region is biased toward task irrelevant region, in agreement to contextual influences that have previously been found in situations where the resolution of sensory inputs was low and thus signals were combined to strengthen the reliability of perception (e.g., Levi et al., 2002; Kersten and Yuille, 2003), or foveal-to-peripheral extrapolation in which foveal vision is used to estimate the strength of stimulus properties in the periphery where visual resolution is limited (Toscani et al., 2017). However, we established the persistence of contextual influences with numerosity information presented for as long as 1 s, indicating that the accuracy of numerical magnitude judgment is not rectified with more processing time added awareness. We suggest that the influence of spatial context and the perceptual bias of perceived numerosity that we observed depends on a top-down mechanism, in which that signals from the irrelevant areas automatically create a perceptual expectation that participants used to infer about their perceived number of dots, because peripheral vision is more limited in terms of acuity than foveal vision. Furthermore, we found this perceptual bias to be affected by top-down contextual factors: the surround effect while judging a central area is most consistent with spatial integration, while the centre effect when judging a surrounding area is better explained as switching between two information channels which we hypothesise to be linked to ambiguous boundary extension. These findings illustrate the complexity and flexibility of processing in peripheral vision (Stewart et al., 2020), and more broadly, our data are in line with evidence for predictive models of vision where top-down priors are combined with incoming sensory inputs (Rao and Ballard, 1999; Friston, 2002; Edwards et al., 2017; Spratling, 2017; De Lange et al., 2018). Peripheral vision is more limited in terms of visual acuity than foveal vision and might involve lower precision predictions than cortical areas processing foveal representations. Nevertheless, our data suggest that peripheral vision encodes its inputs in a context-dependent manner, even when that context is not necessary for the task. This process could serve to explain away information in the periphery during navigation for example, where we could use contextual clues to filter out predictable features that we do not need to attend to. In the future, studies should observe in what measure our results are generalizable to other features and complex displays, potentially introducing multimodal effects. Investigating the neuronal bases of the current findings will also be necessary toward understanding how the visual system encodes numerical magnitude.

## Data Availability Statement

The original contributions presented in the study are included in the article/Supplementary Material, further inquiries can be directed to the corresponding author.

## Ethics Statement

The studies involving human participants were reviewed and approved by the Ethics Committee of the College of Science and Engineering of the University of Glasgow. The patients/participants provided their written informed consent to participate in this study.

## Author Contributions

ML, CA, and LM designed the study together. ML and CA acquired, analysed, and modelled the data. All authors wrote and edited the manuscript.

## Conflict of Interest

The authors declare that the research was conducted in the absence of any commercial or financial relationships that could be construed as a potential conflict of interest.

## Publisher’s Note

All claims expressed in this article are solely those of the authors and do not necessarily represent those of their affiliated organizations, or those of the publisher, the editors and the reviewers. Any product that may be evaluated in this article, or claim that may be made by its manufacturer, is not guaranteed or endorsed by the publisher.
