# Supplementary material for: Numerosity Perception in Peripheral Vision
Source: Front Hum Neurosci. 2021 Nov 3;15:750417. doi: 10.3389/fnhum.2021.750417 (PMC8597708; doi:10.3389/fnhum.2021.750417)

**Supplementary material**


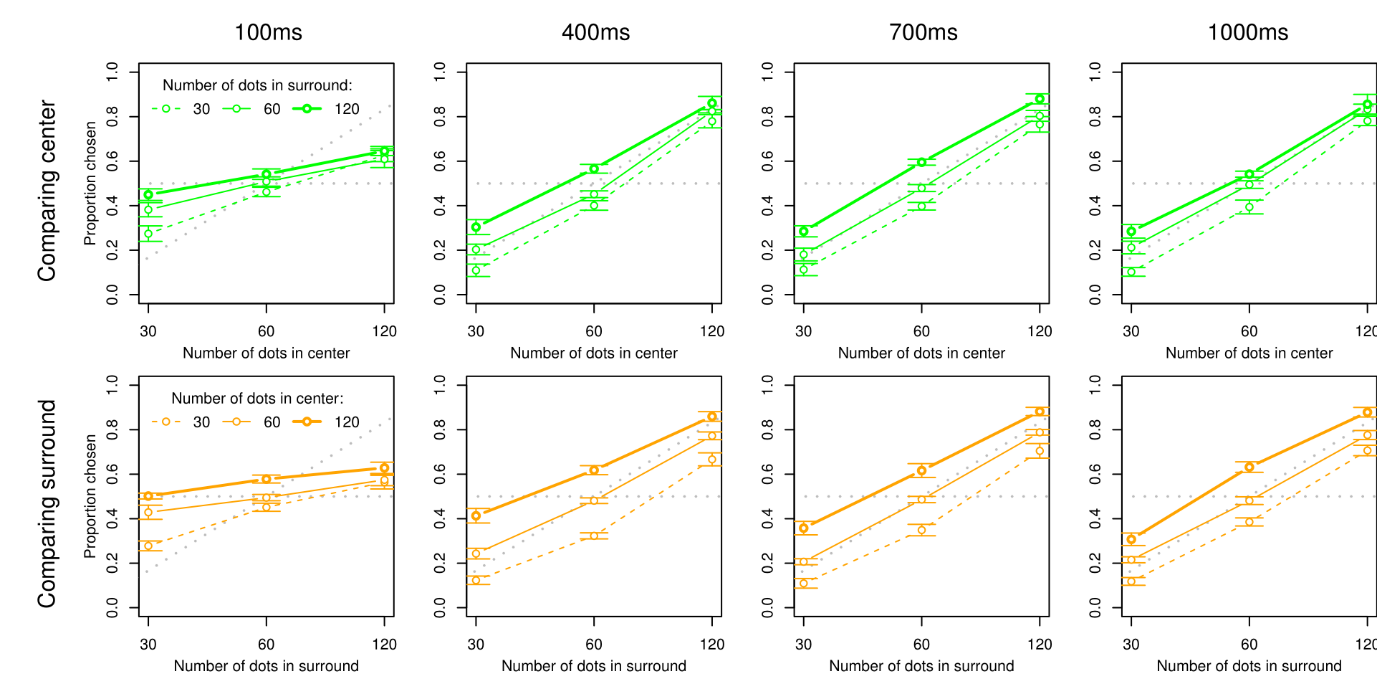


**Supplementary Figure S1**: Proportion of times each combination of center/surround dot number was chosen for both tasks (top row: center task, bottom row: surround task) at each time interval (columns). Within each plot, the number of dots in relevant areas is represented in abscissa while each curve represents the number of dots in irrelevant areas. All error bars are standard error of the mean over participants. Grey lines represent “ideal observers” responding either randomly (horizontal line) or always choosing the highest number of dots in the relevant dimension and randomly when the same number is presented on both sides (this occurs in 1/3 of cases). Note that for the purpose of this analysis each trial is considered twice. For example, in a trial comparing 60 dots in center and 120 dots in surround on the left to 120 dots in center and 120 dots in surround on the right where the observer chose the right side, this would count as a “chosen” trial for the 120 center – 120 surround combination and a “not chosen” trial for the 60 center – 120 surround combination.


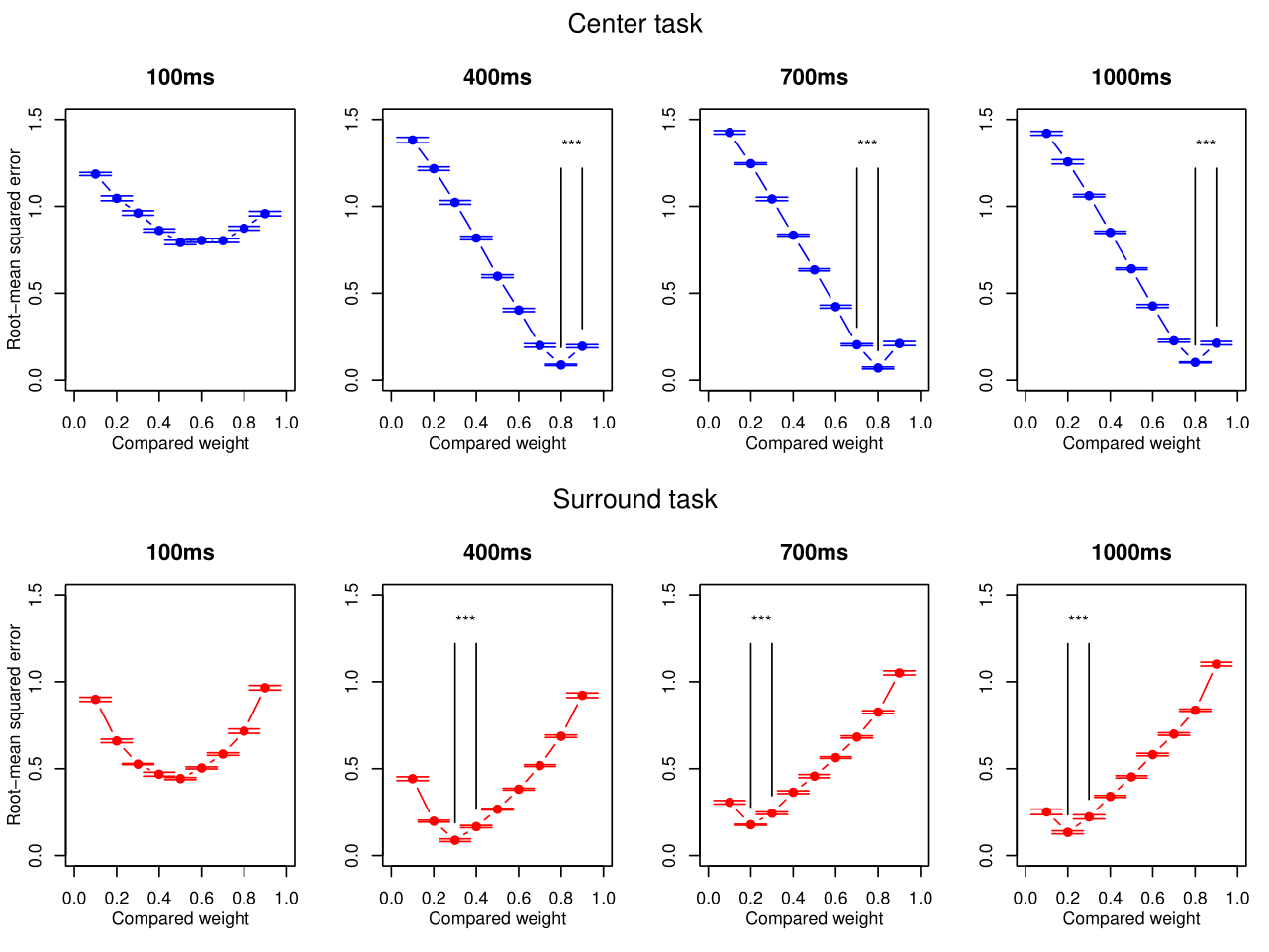


**Supplementary Figure S2**: Illustration of the best weight value (minimal RMSE) for each time interval in both tasks during our simulation analysis. The format is the same as **Figure 2**, bottom row: we represent the distribution of RMSE between our empirical results from 9 observers and 10 simulated datasets, each reproducing all empirical trials, with 9 weights values from .1 to .9, represented in abscissa and corresponding to observer decisions overall being more affected by center (weight closer to 1) or surround (weight closer to 0) information. Top row of panels: Center task results compared to simulated results using the integration model. Bottom row of panels: Surround task results compared to simulated results using the switching model. Each column of panels corresponds to a time interval (100ms, 400ms, 700ms and 1000ms). For each panel, we also represent the comparison between RMSE distributions for the weights corresponding to lowest and second-to-lowest values. Note that we represent only the best decision type (integration or switching) for each task (as illustrated in **Figure 4,** top row), but the weights corresponding to lowest RMSE values for both types are illustrated in **Figure 4**, bottom row, and the comparison between best and second best weights in all cases is detailed in **Supplementary Table S2**.

**Supplementary Table S1**: Testing independent vs additive and additive vs full MLCM models for all conditions, presentation timings and participants.


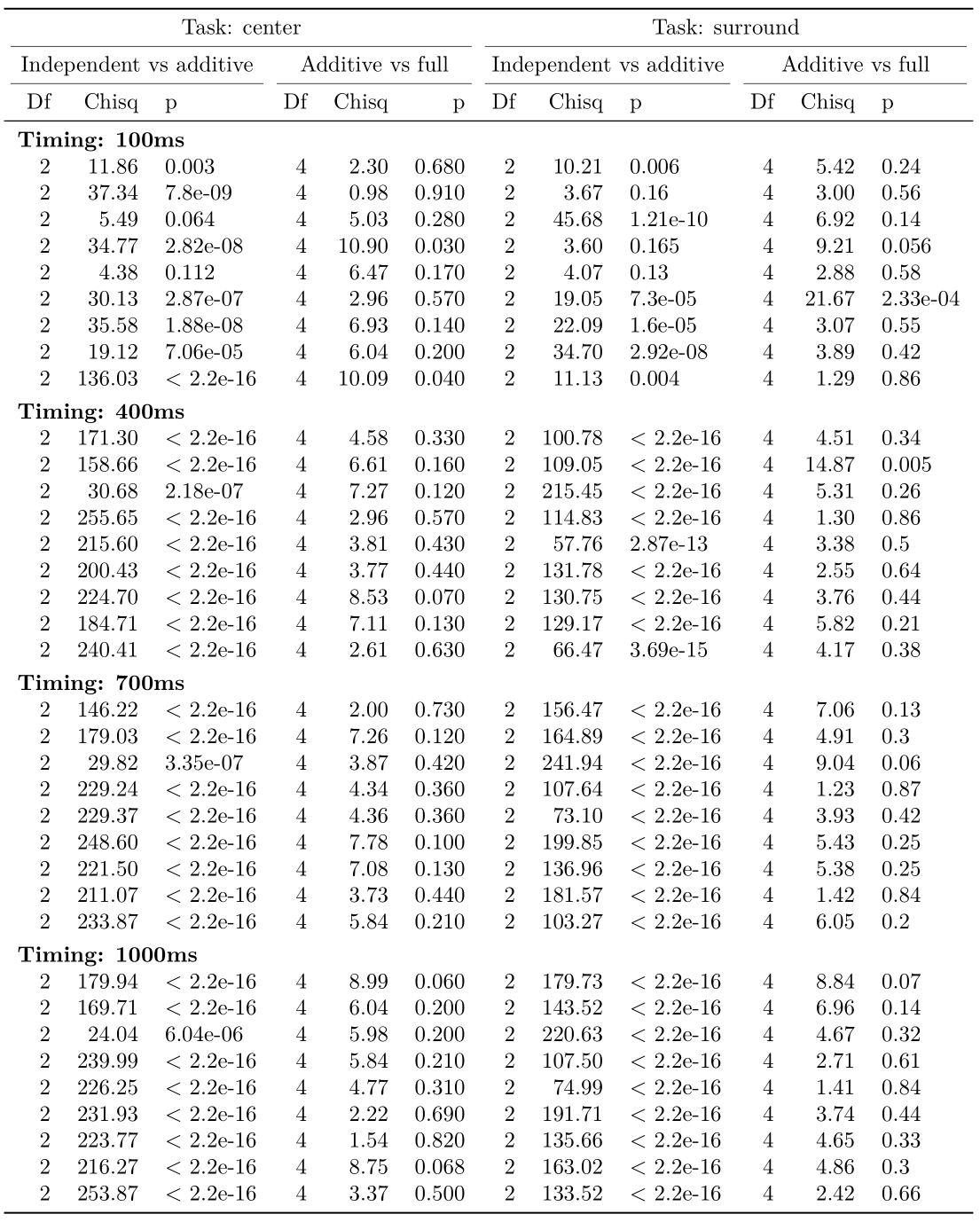


**Supplementary Table S2**: Comparison between RMSE distributions of the weights corresponding to lowest and second-to-lowest values for integration and switching models in all time intervals.


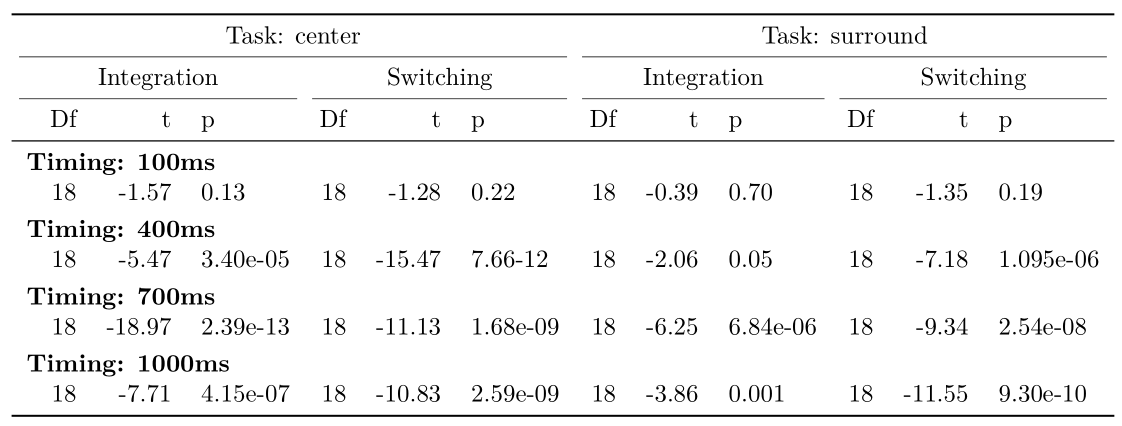

Supplement: Supplementary file 1 [file Data_Sheet_1.docx]
